# Nursing outcomes and social support intervention for diabetes self-management: consensus study

**DOI:** 10.15649/cuidarte.3742

**Published:** 2024-09-01

**Authors:** Claudia Milena Garizábalo-Dávila, Wilson Cañon-Montañez, Alba Luz Rodríguez-Acelas

**Affiliations:** 1 Department of Health Sciences, Universidad de la Costa, Barranquilla, Colombia. E-mail: cgarizab2@cuc.edu.co Department of Health Sciences Universidad de la Costa Barranquilla Colombia cgarizab2@cuc.edu.co; 2 Faculty of Nursing, Universidad de Antioquia, Medellín, Colombia. E-mail: wilson.canon@udea.edu.co Universidad de Antioquia Faculty of Nursing Universidad de Antioquia Medellín Colombia wilson.canon@udea.edu.co; 3 Faculty of Nursing, Universidad de Antioquia, Medellín, Colombia. E-mail: aluz.rodriguez@udea.edu.co Universidad de Antioquia Faculty of Nursing Universidad de Antioquia Medellín Colombia aluz.rodriguez@udea.edu.co

**Keywords:** Self-Management, Social Support, Diabetes Mellitus Type 2, Validation Study, Nursing Outcomes Classification, Autocontrol, Soporte Social, Diabetes Mellitus Tipo 2, Estudio de Validación, Clasificación de Resultados de Enfermería, Autogestão, Apoio Social, Diabetes Mellitus Tipo 2, Estudo de Validação, Classificação dos Resultados de Enfermagem

## Abstract

**Introduction:**

Diabetes mellitus is one of the most prevalent chronic noncommunicable diseases in the world.

**Objective:**

To validate by expert consensus the Nursing Outcomes Classification (NOC) self-management: diabetes (1619) and social support (1504), as well as to validate the intervention of social support for adults in the self-management of type 2 diabetes mellitus (DM2).

**Materials and Methods:**

A consensus study. Several phases were delimited for validation: the first was to validate the results and indicators; the second was to construct and validate the conceptual and operational definitions; and the magnitude of the selected indicators; and the third was to design and validate the intervention of social support for adults in the self-management of DM2.

**Results:**

28 indicators were selected and validated by experts out of the 44 that make up the nursing outcome of self-management: diabetes, and 9 indicators out of the 12 that make up the social support outcome, both with a Content Validity Index (CVI) of 0.98. As for the intervention, a social support intervention was designed for the self-management of DM2, individualized, and made up of 4 sessions. The components of the intervention include generalities of DM2, healthy life habits, safe care, and emotional support.

**Discussion:**

Nursing professionals must evaluate people who experience diabetes, and their capacity for self-management and social support in order to provide appropriate interventions and evaluate their effectiveness.

**Conclusions:**

The study significantly evidenced the validation of the two nursing outcomes and their respective indicators, added to the conceptual and operational definitions, and their magnitude.

## Resumen.

**Introducción:**

La diabetes mellitus es una de las enfermedades crónicas no transmisibles de mayor prevalencia en el mundo.

**Objetivo:**

Validar por consenso de expertos los resultados de enfermería (NOC) de Autocontrol: diabetes (1619) y soporte social (1504), así como validar la intervención de soporte social al adulto para el autocontrol de la diabetes mellitus tipo 2 (DM2).

**Materiales y Métodos:**

Estudio de consenso. Se delimitaron varias fases para la validación: la primera fue validar los resultados e indicadores; la segunda, construir y validar las definiciones conceptuales y operativas; y la magnitud de los indicadores seleccionados; y la tercera, diseñar y validar la intervención de soporte social al adulto en el autocontrol de la DM2.

**Resultados:**

28 indicadores fueron seleccionados y validados por expertos de los 44 que componen el resultado autocontrol: diabetes y 9 indicadores de los 12 que componen el resultado de soporte social, ambos con un Índice de Validez de Contenido (IVC) de 0,98. En cuanto a la intervención, se diseñó una intervención de soporte social para el autocontrol de la DM2, individualizada y compuesta por 4 sesiones. Los componentes de la intervención comprenden: generalidades de la DM2, hábitos de vida saludables, cuidados seguros y apoyo emocional.

**Discusión:**

Los profesionales de enfermería deben evaluar en las personas que viven la experiencia de la diabetes su capacidad de autocontrol y soporte social, con el fin de proveer intervenciones apropiadas y evaluar su eficacia.

**Conclusiones:**

El estudio evidenció significativamente la validación de los dos resultados de enfermería y sus respectivos indicadores, sumados a las definiciones conceptuales y operacionales y su magnitud.

## Resumo.

**Introdução:**

O diabetes mellitus é uma das doenças crônicas não transmissíveis mais prevalentes no mundo.

**Objetivo:**

Validar por consenso de especialistas os Resultados de Enfermagem NOC Autogestão: diabetes (1619) e Apoio social (1504); bem como validar a intervenção de apoio social para adultos para a autogestão do diabetes mellitus tipo 2 (DM2).

**Materiais e Métodos:**

Estudo de consenso. Foram delimitadas várias fases para validação; a primeira foi a de validação dos resultados e indicadores; a segunda, construir e validar as definições conceituais e operacionais e a magnitude dos indicadores selecionados; e na terceira, projetar e validar a intervenção de apoio social para o adulto na autogestão do DM2.

**Resultados:**

Foram selecionados e validados por especialistas 28 indicadores dos 44 que compõem o resultado autogestão de enfermagem: diabetes; e 9 indicadores dos 12 que compõem o resultado apoio social, ambos com Índice de Validade de Conteúdo (IVC) de 0,98. Quanto à intervenção, foi elaborada uma intervenção de apoio social para autogestão do DM2, individualizada, composta por 4 sessões, os componentes da intervenção incluem, generalidades do DM2, hábitos de vida saudáveis, cuidado seguro, apoio emocional.

**Discussão:**

Os profissionais de enfermagem devem avaliar as pessoas que vivenciam o diabetes, sua capacidade de autogestão e apoio social, para fornecer intervenções adequadas e avaliar sua eficácia.

**Conclusões:**

O estudo evidenciou significativamente a validação dos dois resultados de enfermagem e seus respectivos indicadores, somados às definições conceituais, operacionais e sua magnitude.

## Introduction

The International Diabetes Federation (IDF)^1^ has estimated that there are currently 537 million adults with diabetes worldwide, and it is projected that by the year 2045, this number will have increased to 783 million. More than 90% of these cases correspond to type 2 diabetes mellitus (DM2).

In 2021, it was possible to demonstrate that 44.7% of adults living the experience of diabetes mellitus (DM) were unaware of their health status. This delay in the time of diagnosis leads to a high risk of suffering from complications that affect their quality of life not only from the biological perspective but also from the psychological and social^2^^, ^^3^, creating an overload in the health care system^4^.

The scientific literature evidences that social support is a factor that influences diabetes self management behaviors, as referenced by the middle-range nursing theory of Individual and Family Self-management^5^^, ^^7^.

Pertinent also is the implementation of effective nursing interventions for self-management and social support, along with indicators that allow nurses to assess the health status and sensitivity to care interventions that lead to decision-making that favors the well-being of individuals experiencing DM2 and involve their family environment.

In this regard, nursing professionals play a fundamental role in supporting people who experience this condition. They assess the adult according to their needs and own context, using outcome labels that establish parameters for evaluation, analysis, and informed decision-making in the health process, while implementing nursing interventions that contribute to the acquisition of self-management skills. Furthermore, they promote healthy lifestyles and contribute to the management of complex treatment regimens, empowering adults to control their own health and realizing social support^8^^, ^^9^.

The Nursing Outcomes Classification (NOC) provides labels that allow a comprehensive assessment of individuals with DM in response to a nursing intervention. Among the outcomes that weigh adult diabetes self-management is “Self-management: Diabetes” (1619), defined as "Personal actions to manage diabetes, its treatment, and to prevent complications". This NOC outcome has 44 indicators to assess self-management behaviors and was introduced in the fifth edition (2013) ^10^. The second outcome is “Social Support” (1504), defined as "reliable assistance from others", which comprises 12 indicators^11^.

Although the NOC “Self-Management: Diabetes” (1619) was validated in a previous study, this validation was conducted in English and within a North American population. As a result, it as recommended to validate it in other languages and particular cultures^9^. For its part, the outcome “Social Support” (1504) was not found in validation studies in the literature.

Building preliminarily on the NOC outcomes “Self-management: diabetes” (1619) and “Social support” (1504), the paucity of social support nursing interventions for diabetes self- management^12^, the need for measurement instruments corresponding to nursing interventions in this context, and the existing nursing theoretical support for developing interventions targeting this research phenomenon^7^, the objective of this study was to validate, by expert consensus, the NOC “Self management: diabetes” (1619) and “Social support” (1504). Additionally, the study aimed to construct the conceptual and operational definitions of their respective indicators and to design and validate an intervention of social support for adults in the self-management of DM2.

## Materials and Methods

### Study design

This is a methodological consensus study conducted in two sequential phases between March 2020 and May 2021 in Colombia. The first phase comprised the expert consensus validation of the NOC Self Management: Diabetes (1619) and NOC Social Support (1504) ^11^ and their respective indicators, along with conceptual, operational and the magnitude. The second phase involved designing and obtaining expert consensus validation for the adult social support intervention for DM2 self-management.

### Sample

Nine experts participated in the first phase of the study meeting the following criteria: being a nursing professional with over two years of experience in the area of chronic noncommunicable diseases, having scientific production on nursing process and standardized language systems, and having research and/or academic experience in the area. The number of experts was determined by convenience based on previous studies^13^^, ^^14^.

For the second phase of the study, the sample was composed of eight experts and delimited according to previous studies and fulfilling the following inclusion criteria^15^^, ^^16^: being a health professional with over two years of experience in the management of chronic noncommunicable diseases and having scientific production on or research and/or academic experience in the management of health interventions.

### Data collection

Nursing professionals were recruited through email invitations and recommendations from the participants themselves, with this phase being conducted entirely online. The first phase contemplated the characterization of the population, general guidelines to facilitate its development, information on the NOC outcome labels “Self-management: diabetes” (1619) and “Social support” (1504)^11^, and their respective indicators, with "selected and not selected" response options, and an additional space for comments.

Once the outcomes and indicators were selected by the experts, the researchers proceeded to construct the conceptual and operational definitions for each indicator and its magnitude, with the above being based on the literature^17^^, ^^18^ and experience of the researchers. Next, the material was sent, where the experts had three response options for the validation of the information: agree, agree with modifications, or disagree. With this part, the first phase was completed.

The second phase of the study comprised the design and validation by consensus of specialists of an adult social support intervention for self-management of DM2, which was developed based on the results of phase I and under the guidelines of Sidani and Braden^19^. This includes: a) a clear and complete understanding of the problem, describing the nature, manifestations, level of severity, causal factors, and consequences of the problem; b) an elaboration of the intervention delimited by the scientific literature and the medium-range nursing theory; c) the development ofthe intervention based on the results of phase I and under the guidelines of Sidani and Braden^19^^, ^^22^ and the mid-range nursing theory called "Individual and Family Self-Management Theory" by Ryan^7^.

From these aspects, the researchers developed the preliminary version of the intervention, which led to the validation by expert consensus of the intervention content, allowing correspondence between elements of the intervention.

The content of the intervention was sent by means of an instrument that included informed consent, characterization of the participants, guidelines for the validation of the content of the intervention, and a link that allowed them to access the intervention in its entirety.

Participants assessed the correspondence between the active principles, components, activities, and actions of the intervention through the consistency and completeness of the components and their relationship to the active ingredients of the intervention, an analysis of the appropriateness of activities to operationalize the components, examining the accuracy and relevance of point and non-specific actions in the delivery of the intervention activities; determining any omissions of components, activities, and actions; and suggesting ways to revise the intervention elements to improve the correspondence between them^19^.

This validation of the intervention ended with two rounds, using three criteria: agree, agree with modifications, and disagree, plus a box for comments.

### Data Analysis

A Content Validity Index (CVI) > 0.80 was defined for the study of each of the two phases^23^. The Survey Monkey Audience program was used for data collection, and the synthesis of the content was standardized using the Windows Excel program. The database was stored in Mendeley Data^24^.

### Ethical criteria

The study was approved by the Research Ethics Committee of the institution involved (CEI-FE 2020 04). All participants approved the informed consent online.

## Results

### Socio-demographic characterization

For the first phase of the study of the 9 experts who participated in the validation of the labels, there was a predominance of the female gender (n= 8, 89%), with an average age of 44 years. Regarding the level of education, 8 were identified as having a master's degree (89.00%), and 1 expert with a doctoral degree (11.00%). Regarding professional experience, this group of experts has more than 20 years in the area of study.

In the second phase of the 8 experts, the majority were female, with an average age of 42 years; 100% nursing professionals, 75.00% (n= 6) with master's degrees and 25.00% (n= 2) with a doctoral degree; with work experience in the context of the study of more than 20 years.

### First Phase:

The NOC Social Support (1504) was validated by experts with 9 indicators from the 12 that compose it, which are listed in Table 1.

**Table 1:** Selected Social Support Outcome Indicators.

NOC Label -Social Support (1504)
(150408) Willingness to call on others for assistance
(150412) Assistance offered by others
(150402) Time provided by others
(150403) Refers to tasks performed by others
(150404) Refers to information provided by others
(150405) Refers to emotional support provided by others
(150406) Refers to trust relationships
(150407) Refers to the existence of people who can help you when you need it
(150409) Refers to a social support network

*NOC: Nursing Outcomes Classification*

The indicators “supportive social contacts” (150410) and “stable social network” (150411) were excluded, since the experts considered that they present similarities between them which could generate a bias at the time of data collection^14^. This is due to the fact that the indicator Refers to a social support network (150409) was included, and this also has similarities. Finally, the indicator Refers to economic help from other people (150401) was excluded, as the experts stated that it did not respond to the intervention of a nursing professional, but rather pointed more to a family type of support.

The Nursing Self-Management Outcome: Diabetes (1619) is made up of 44 indicators, 28 indicators were selected and are listed in Table 2.

**Tabla 2:** Selected indicators of the Self-Management Outcome: diabetes.

NOC Label - Self-management: diabetes (1619)
(161901) Accepts diagnosis
(161902) Seeks information on methods to prevent complications
(161903) Performs preventive foot care practices
(161906) Reports non-healing skin lesions to the primary care practitioner
(161907) Participates in health care decisions
(161908) Participates in prescribed educational program
(161909) Performs treatment regimen as prescribed
(161910) Performs the correct procedure for blood glucose monitoring
(161911) Monitors blood glucose
(161915) Reports symptoms of complications
(161916) Uses a diary to monitor blood glucose over time
(161917) Uses preventive measures to reduce the risk of complications
(161941) Obtains health care if blood glucose fluctuates outside of recommendations
(161920) Follows the recommended diet
NOC Label - Self-management: diabetes (1619)
(161921) Follows recommended activity level
(161922) Monitors body weight
(161923) Uses effective weight control strategies
(161925) Follows the recommendations for alcohol consumption
(161926) Participates in a smoking cessation program
(161929) Uses the correct procedure for insulin administration
(161930) Stores insulin correctly
(161931) Obtains necessary medication
(161932) Uses medication as prescribed
(161933) Monitors medication therapeutic effects
(161934) Rotates injection sites
(161937) Uses health care services congruent with needs
(161939) Keeps appointments with health professional
(161940) Maintains plan for medical emergencies

*NOC: Nursing Outcomes Classification*

The experts made some considerations regarding the excluded indicators. For instance, Controls glycosuria and ketonuria was excluded because its measurement is complex for the study requiring control by clinical laboratories. Uses only non-prescription medications approved by health professional was considered complex to interpret and may not be easily understood by patients, as self-medication has serious health implications. Obtains the pneumonia vaccine, although relevant in the context of diabetes, requires a shared decision-making discussion with their treating physician to determine the individualized risks and benefits^17^. Finally, Monitor for signs and symptoms of depression was analyzed as a parameter that requires interdisciplinary management for patient monitoring and would not apply to the entire study population. A CVI of 0.98 was obtained among the experts.

Once the indicators were selected, we proceeded to develop the conceptual and operational definitions and the magnitude of the nursing outcomes.

### Second Phase:

The intervention was developed on the basis of the steps proposed by Sidani and Braden^19^ which are mentioned below:

### Step 1: Clear and complete understanding of the problem

A theoretical and an empirical approach was used, which allowed for greater clarity on the nature, manifestations, level of severity, causal factors, and consequences of the problem (see Table 3). At the theoretical level, the referent was the Theory of Individual and Family Self- Control, which considers the individual and their family^7^, as well as their context. This theory defines self-management as the intentional incorporation of health-related behaviors in the daily functioning of an individual, preventing diseases or facilitating the management of complex health regimens, reflecting individual and family values and beliefs in a meaningful way.

At the empirical level, the scientific literature provided a relevant information on the prevalence, manifestations, level of severity, determinants and factors associated with the study phenomenon, allowing for expanded understanding of the problem^23^^, ^^25^.

**Table 3:** Presentation of the problem to be intervened in adults for DM2 self-management.

Nature of the problem	Self-management is conceptualized as the ability of the individual, together with the family, community and health professionals, to manage symptoms, treatments, lifestyle changes, and psychosocial, cultural, and spiritual consequences of health conditions.
Manifestations of the problem	People with DM2 may present alterations in metabolic parameters, persistent symptoms, low therapeutic adherence, complications, frequent hospitalizations, increased costs associated with the use of care, and risk of mortality. Likewise, and in relation to other domains, they may present a lack of knowledge of the disease, a lack of autonomy in health care, a lack of participation and skills in preventive health behaviors, and finally, from the social domain, difficulties in decision- making and problem-solving, lack of motivation to control their disease, alterations in coping, lack of social acceptance, difficulty in processing emotions, and loss of productivity.
Level of severity of the problem	The lack of self-management behaviors contributes to the progression of the disease, leading to health complications, decreased functional capacity, and a lower quality of life.
Causal factors of the problem	-Personal characteristics and lifestyle: knowledge, health, cultural and spiritual beliefs, life patterns, psychological disorder, motivation -Health status: genetics, comorbidity, severity of disease and symptoms. -Resources: financial, psychosocial devices and resources. -Environmental characteristics: environments, home, work and community. -Health care system: access to health services. -Individual and family factors: age, culture, psychosocial characteristics, family functionality .
Consequences of the problem	The lack of control over DM2 and insufficient participation in health-promoting lifestyles increases the risk of mortality and lead to serious biological, psychosocial and economic complications for the individual, their family and the healthcare system.

*DM2: Type 2 diabetes mellitus.*

### Step 2: Identification of the aspects of the problem that can be changed

It was supported based on three assumptions of the Individual and Family Self-ManagementTheory^7^: 1) social support can direct, encourage and support engagement in self-management behaviors and the achievement of outcomes; 2) person-centered interventions are more effective in fostering engagement in self-management behaviors and achieving of outcomes; and 3) individuals actively participate in self-management conditions by collaborating with people in the health care system to achieve personal health goals.

### Step 3: Outlining intervention strategies

Based on the aspects of the problem identified in the previous steps, strategies were described which in turn form active ingredients of the intervention^19^: knowledge of DM2, DM2 self-management skills, and social support.

Steps 4-5: Select the mode of administration and dose of the intervention; specify the elements of the intervention

These steps were constructed taking into account the scientific literature and taking into account the active ingredients of the intervention which are described in general terms in Table 4.

**Table 4:** Specific elements of the intervention.

Name of intervention	Social support intervention for DM2 self-management.
Dose	-4 sessions
	-Once every 15 days for 2 months -2 follow-up sessions at 1 month and 2 months after the intervention
Duration	40 minutes
Delivery strategies	-Individualized -Face to face -Use of educational material (educational brochure)
Place of delivery	Nursing consultation (outpatient clinic) from a health institution level of care
Receiver	Adults with type 2 diabetes mellitus who meet the inclusion criteria
Provider	Nurse professionally trained in the management of type 2 diabetes mellitus

*DM2: Type 2 diabetes mellitus.*

### Validation of the intervention

Once the intervention had been constructed following Sidani & Braden^19^, it was validated by eight experts, obtaining an CVI of 0.90; among the disagreements, one expert recommended that the place of delivery should contemplate not only the nursing office, but also the individual's home; in addition, that the provider should include an interdisciplinary health team, which was not accepted because the intervention focuses on the role of nursing in the care of DM, in the primary health care nursing consultation.

Regarding the precision, feasibility, acceptability of the intervention, coherence and completeness between the components and principles of action, appropriateness of the activities, and precision and relevance of the actions, 100% of the experts agreed with the elements that make up the intervention protocol. However, the experts recommended specifying the actions and establishing specific differences between activities and actions to avoid similarities.

In response to these recommendations, the respective adjustments were made and the protocol was sent for a second round, which did not generate any further recommendations. The CVI achieved was 0.98.

## Discussion

The study allowed validation of the NOC Self-management: diabetes (1619) and Social support (1504), as well as the validation of the adult social support intervention for self-management of DM2, based on a middle-range nursing theory and scientific literature. Within the Nursing Outcome NOC Social support (1504), the indicators: refers information provided by others (150404) and refers the existence of people who can help you when you need it (150407) indicated a higher CVI. This is relevant, since in correlation with the literature, the first indicator is based on the fact that adequate social support facilitates the necessary knowledge through the provision of information by a nursing professional^17^. Likewise, the second is seen as essential because of the role that nursing influences the achievement of health outcomes in patients^17^^, ^^26^.

Regarding the NOC Self-management: diabetes, of the 28 indicators selected as relevant in nursing practice, the indicators Seeks information on methods to prevent complications (161902) and Reports symptoms of complications (161915) obtained 100% consensus. The aforementioned is related to the identification of the educational needs of patients against knowledge and awareness of the complications of diabetes to improve the ability to control the disease and decrease complications^27^. This finding coincides with another study^9^.

For the Participate in health care decisions indicator (161907), the national standards for diabetes self-management education and support recommend that all people with diabetes, to the extent possible, participate in decision-making of informed decisions about their health, in addition to having skills that lead to diabetes self-management^17^.

For the indicator Participates in prescribed educational care (161908), the literature refers to the necessity of the individual to recognize the therapeutic objectives that lead him/her to achieve self management of DM^17^.

For Uses medication as prescribed (161932), this decision about which medications are best for patients are determined by the health care provider and depends on many factors, including blood glucose level, symptoms, and other health problems presented^28^.

Uses health services congruent with needs (161937) and keeps appointments with health professional (161939) were considered fundamental indicators, because the person with DM should see his or her health care team continuously and this is emphasized in people who have problems reaching blood glucose, blood pressure or cholesterol targets, since care with health professionals promotes prevention and treatment of any health problem^17^.

For Reports non-healing breaks in the skin to the primary care professional (161906), skin manifestations of DM allow patients to provide information on skin health status, also becoming a marker of glycemic control, making this indicator relevant^29^.

Regarding the intervention, commonalities and differences were found. Reviewing the components, the social support intervention designed in the present study comprised informational support, which encompasses DM generalities and healthy lifestyle habits; instrumental support containing safe care; and emotional support.

These components were designed following the guidelines of the American Diabetes Association (ADA), the International Diabetes Federation, and the Nursing Theory of Individual and Family Self- management^7^, and other literature references.

Common to the studies reviewed are McEwen et al^30^, who considered nutrition, physical activity, prevention of complications and management of distress as fundamental components of the study. These coincide with some of the components of this one, such as healthy lifestyle habits, related to nutrition, which include label reading and identifying healthy foods in daily meal menus, among others. Likewise, other studies were acknowledged that in their interventions contemplated this component of healthy lifestyle habits^31^. However, the present study considers more broadly the component of healthy lifestyle habits, including the management of alcohol and tobacco, aspects recommended by the ADA^17^.

Another essential component contemplated by the social support intervention for the achievement of diabetes self-management is to provide informational support related to the generalities of diabetes, seeking that those experiencing DM2 know the disease process, follow complex treatment regimens, monitor their condition, make lifestyle changes, and make decisions to manage health problems^9^. With respect to this component, few studies have contemplated these aspects^27^^, ^^30^.

On the other hand, regarding the instrumental support component, which comprises safe care with topics such as symptom management, glucose monitoring, pharmacological treatment management and foot care, some aspects are considered by other interventions that seek the same objective of achieving DM2 self-management^31^. However, some of these interventions emphasize pharmacological management and other biological variables such as glycemic control, lipid control, Body Mass Index, among others, which could be interpreted as interventions for the self-management of DM2^21^^, ^^31^. This could be interpreted as interventions in a biomedical or biologicist model, which does not include such necessary aspects as emotional support^17^.

Regarding social support, it was considered for the study as an essential component for the achievement of DM2 self-management, given that psychological and social problems can affect the individual's ability to perform diabetes care tasks, and thus potentially compromise health status^17^. Consistently, the social support intervention comprises a special module on social support, which includes emotion management, communication and decision-making, empowerment and confidence, and support groups or networks.

Little evidence was found of interventions that contemplate the social support provided by health professionals. The study by Samuel-Hodge et al^32^ included stress and conflict resolution in their intervention; in addition, included problem solving and decision making. Grey et al^33^ proposed a psycho-educational program based on the Internet, where they addressed stress, coping, self-control behaviors, self-efficacy, social competence, and family conflicts.

Although this is an intervention aimed at a different type of population, there are very relevant aspects in common.

This similarity between the components of the interventions is consistent with the theory that guides the study "Theory of Individual and Family Self-Control"^7^ which states that people living a situation of chronicity such as DM2 should be addressed with interventions that contain knowledge and beliefs, self-management behaviors (nutrition and physical activity), decision-making and social support. When reviewing the professional who performs the interventions in studies similar to this one, most of them are performed by nurses, which highlights the crucial role of nurses in supporting people living with DM2^30^.

On the other hand, with regard to the number of sessions provided in the interventions, a review of literature reports between 3 and 6 sessions^30^. One of these studies presented 6 sessions during 6 months, 5 sessions were developed in the first 3 months, and the last session was offered by telephone calls. Others relating to these interventions were developed weekly during the first three months, whereas in the remaining three months the sessions were carried out with time intervals of 15 days between them^33^.

The McEwen study^30^ consisted of 9 sessions, with 6 being group sessions and 3 being individual. During the first month, the frequency of the intervention was one week, after the first month it was increased to two per week, until completing a total of 3 months. These results, when compared with those obtained in this study, show that the number of sessions considered in the social support intervention are within the limits of the sessions developed by other studies (between 3 and 9). However, the intervals between sessions are longer meaning it usually takes more time to develop an intervention.

This situation may be due to the fact that the contexts in which the interventions are developed have a different health system and have funding for research.

Finally, nursing professionals play an important role in clinical and primary care research and practice regarding the design, implementation, and evaluation of effective and innovative interventions for DM2 self-management; thus, nursing interventions that seek to improve DM2 self-management should consider including these types of key aspects for goal attainment^17^.

## Conclusion

This study allowed the selection and validation of the Nursing Outcomes: Self-Management: diabetes and Social Support with their respective indicators, in addition to the construction of conceptual definitions, operations and magnitude, which will allow the evaluation of patients in search of self-management of DM2 using a standardized classification, since the relevance indexes of these outcome indicators met the criteria for categorization as fundamental and sensitive indicators. This way, the study contributes to the scientific literature, a set of conceptual and operational definitions of the outcome indicators, Self-Management: Diabetes, and Social Support; that will facilitate the use of a standardized nursing language in the context of practice, to strengthen the nursing process in the management of people who live the experience of Diabetes.

Similarly, the development and validation of the nursing intervention of social support in the adult for the self-management of DM2 based on scientific evidence and the theory of mid-range nursing will enable all health professionals to have a guide for nursing consultations to the patient with DM2, aiming at the comprehensive management of the dimensions of the human being.
